# New Dental School in New York

**Published:** 1893-02

**Authors:** 


### NEW DENTAL SCHOOL IN NEW YORK.

The announcement has been made of the establishment of a new College of Dentistry in New York City "for men and women."

It has been a foregone conclusion for some time that New York would have the second school, and it is rather remarkable that this has not been established before.

It starts out on a liberal basis, and for this the incorporators are to be congratulated.

The Faculty has not been announced, but we understand that Dr. W. F. Rehfuss, of Philadelphia, is to have the chair of Dental Jurisprudence. The selection is the best that could have been made, and gives promise of a strong staff of workers in the several departments.

